# A Rapidly Growing Nodule on the Eyebrow of a Pediatric Patient

**DOI:** 10.3390/dermatopathology11040028

**Published:** 2024-09-30

**Authors:** Italo Francesco Aromolo, Michela Brena, Nicola Adriano Monzani, Fabio Caviggioli, Emilio Berti, Donata Micello, Riccardo Cavalli

**Affiliations:** 1 Department of Pathophysiology and Transplantation, Dermatology Unit, University of Milan, 20122 Milan, Italy; 2 Pediatric Dermatology Unit, Department of Clinical Sciences and Community Health, Foundation IRCCS Ca’ Granda Ospedale Maggiore Policlinico, 20122 Milan, Italy; 3 Department of Clinical Science and Community Health, University of Milan, 20122 Milan, Italy; 4 Plastic Surgery Unit, Istituto di Ricovero e Cura a Carattere Scientifico (IRCCS) MultiMedica, 20123 Milan, Italy; 5 Inter-Hospital Division of Pathology, Istituto di Ricovero e Cura a Carattere Scientifico (IRCCS) MultiMedica, 20138 Milan, Italy

**Keywords:** pediatric dermatology, cutaneous neoplasm, dermatopathology

## Abstract

A 11-year-old Caucasian girl presented to our Dermatology Unit with a 2-month history of an erythematous nodule, localized to the medial portion of her left eyebrow, rapidly growing in the two weeks before presentation. The histopathological examination revealed a dermal multi-nodular epithelial neoplasm composed of clear cells, squamous cells, and glandular cells, characterized by cytologic atypia, high mitotic activity, and an infiltrative deep growth pattern. The immunohistochemical profile of the lesion was as follows: CKAE1/AE3+, EMA+, CK8/18+, CK7+, CK19+, AR negative, p63 focally +, Ki67 25%, rare cells GCDFP15+, p53+.

## 1. Introduction

**Figure 1:**
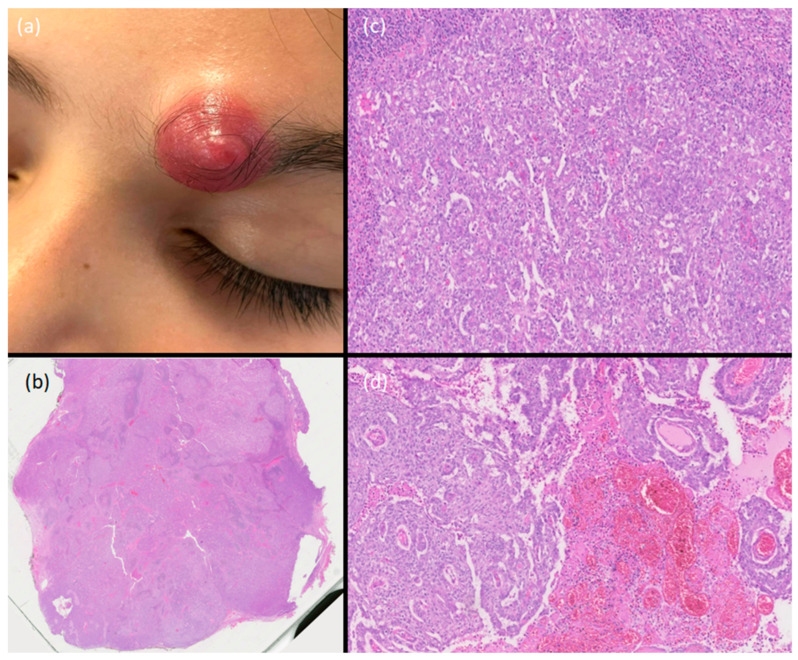
Erythematous nodule at the medial root of the left eyebrow arch (**a**). A dermal multi-nodular epithelial neoplasm (H&E, 0.5×) (**b**). An infiltrative growth pattern with clear cells and duct-like glandular structures (**c**) and squamous cells with hemorrhages (**d**) (H&E, 12× and 10×).

A 11-year-old Caucasian girl attended our Dermatology Unit with a 2-month history of an erythematous nodule, hard to elastic on palpation, without a central opening, approximately 15 mm in size, localized to the medial portion of her left eyebrow, rapidly growing in the two weeks before presentation. No other dermatological lesions were detected before it appeared. On dermoscopy, a nonspecific vascular pattern was appreciable. The patient had already been treated with systemic amoxicillin/clavulanic acid for 8 days, without improvement. Her past medical history was unremarkable. A skin ultrasound showed a round formation approximately 12 × 9 mm, involving the dermis with subcutaneous extension and a rich perilesional vascularization. The lesion had sharp margins, with posterior acoustic enhancement, and an ecostructure mainly solid and partly liquid. We decided to perform a surgical excision of the lesion, which meanwhile continued to grow (Figure 1a).

**Figure 2:**
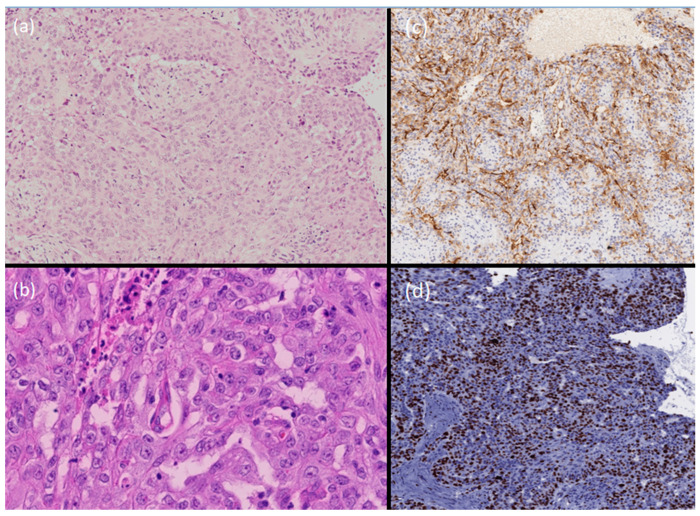
Infiltrative neoplastic growth composed of atypical cells, sometimes lining tubular structures. Areas of squamoid differentiation are appreciable (H&E, 16×) (**a**). A detail where clear cells are appreciable (H&E, 20×) (**b**). EMA staining highlights ductal structures (EMA, 12×) (**c**). Ki67 is about 25% in the “hotspot” areas (Ki67, 15×) (**d**).

The histopathological examination revealed a dermal multi-nodular epithelial neoplasm composed of clear, squamous, and glandular cells, characterized by cytologic atypia, high mitotic activity, and an infiltrative deep “top–down” growth pattern, with hemorrhages. A reactive lymphoplasmacytic infiltrate with eosinophils was appreciable (Figure 1b–d and Figure 2a,b). The luminal borders of the ductal structures were highlighted by EMA staining (Figure 2c), and the Ki67 positivity rate was estimated to be around 25% in “hotspot” areas (Figure 2d).

**Figure 3:**
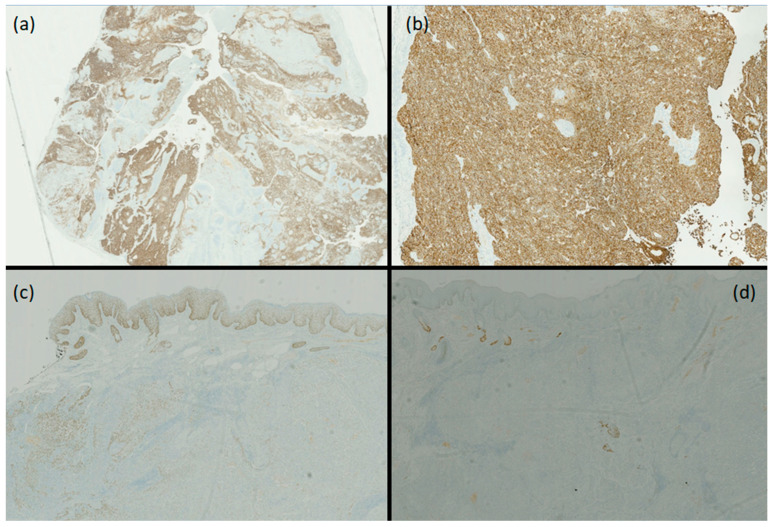
CK7 is positive (**a**) and CK8/18 is also positive (**b**), confirming adnexal differentiation. p63 is positive only in areas with squamoid differentiation but negative in most of the neoplasm (**c**). The androgen receptor is negative (**d**). Magnification: (**a**) 1×; (**b**) 5×; (**c**) 2.5×; (**d**) 2.5×.

The complete immunohistochemical profile of the lesion was as follows: CKAE1/AE3+, GATA3+ (suggesting apocrine differentiation), EMA+, CK8/18+, CK5+, CK7+, CK19+, CA125 focally +, EpCAM focally +, AR negative, p63 focally +, Ki67 25%, rare cells GCDFP15+, p53+, S100−, BCL2−, WT1−, TG-, TTF1−, PAX8−, DOG1−, CD117−, vimentin−, synaptophysin−, CK20−, CDX2−, alpha inhibin−, ER−, PR−, and EBV− (Figure 3).

## 2. What Is the Diagnosis?

(a) Hidradenoma;
(b) Hidradenocarcinoma;
(c) Clear-cell squamous cell carcinoma;
(d) Porocarcinoma;
(e) Trichilemmal carcinoma.

## 3. Diagnosis

(b) Hidradenocarcinoma.

## 4. Discussion

Hidradenocarcinoma is a rare malignant and aggressive tumor of the sweat glands, with an estimated incidence of <0.05% [1,2]. It is more frequent in females between 50 and 70 years of age. Pediatric cases are exceptional [2,3]. It commonly presents as a rapidly growing subcutaneous nodule, frequently located on the scalp and face. Most cases arise de novo, but some can develop from pre-existing hidradenomas. Clinical differentation between hidradenoma and hidradenocarcinoma is challenging [2].

Histologically, hidradenocarcinoma appears as a multilobulated dermal neoplasm, without significant epidermal connection, composed of a mixture of eosinophilic polygonal cells, squamous cells, clear cells, and mucinous cells, sometimes lining tubular/ductal structures. In contrast to its benign counterpart (hidradenoma), hidradenocarcinoma presents with an infiltrative growth pattern, deep extension, nuclear pleomorphism, areas of necrosis, ≥4 mitoses per high-power field, and a Ki67 > 11%, as in our case [4]. The presence of focal atypical features in otherwise benign-appearing tumors complicates the differential diagnosis, identifying the so-called atypical hidradenoma [4]. The other differential diagnoses included other malignancies, especially adnexal (sebaceous carcinoma, adenoid cystic eccrine carcinoma, eccrine adenocarcinoma, mucinous eccrine carcinoma, porocarcinoma, and trichilemmal carcinoma) but also clear-cell squamous cell carcinoma and metastases [5]. Metastasis was ruled out for a “top–down” pattern of histological involvement with the presence of well-differentiated areas, and the negativity of organ-specific immunohistochemistry. The porocarcinoma was excluded based on cellular morphology (absence of poroid cells); the clear-cell squamous cell carcinoma and the trichilemmal carcinoma were excluded due to the absence of epidermal connection, the presence of duct-like structures, and based on the immunohistochemistry panel (CK7+, CK8/18+; EpCAM and p63 only focal). The therapy of choice is surgical excision with wide margins, given the high local recurrence rates in about 50% of cases. Some reports suggest adjuvant chemotherapy and/or radiotherapy [5]. Data on the prognosis of hidradenocarcinoma are partially conflicting. According to some papers, there is a 60% risk of metastasis within 2 years of diagnosis (to lymph nodes, lungs, and bone), with a 5-year survival rate of 30% [1]. In a large case series of 289 patients, the prognosis is relatively favorable, with 10-year overall and cancer-specific survival rates of 60.2% and 90.5%, respectively [6]. A reason for this discrepancy could be diagnostic bias, as the clear-cut histologic distinction between atypical hidradenoma and hidradenocarcinoma may be challenging [4].

In our case, no nodal or metastatic localization was detected at positron emission tomography and magnetic resonance staging. After 1 year of clinical and radiological follow-up, without other therapies, no local recurrences or distant metastases were observed.

Two other cases of pediatric hidradenocarcinoma have been described, both retroauricular in location: one was skin-limited and free from disease with surgical excision alone after one year of follow-up [2]; the other also involved local lymph nodes, and the patient was alive after surgery (including total lymphadenectomy) and adjuvant radiotherapy [3]. In our opinion, the present case is of interest because it presents a rare condition, typical of the adult population, observed in a young adolescent, with an apparent good prognosis. Considering the scarcity of pediatric cases, further data will be necessary to better characterize this population.

## Data Availability

Anonymized data will be shared upon reasonable request from any qualified investigator for the purposes of replicating the procedures and results.
